# Sensors for Human Physical Behaviour Monitoring

**DOI:** 10.3390/s23084091

**Published:** 2023-04-19

**Authors:** Malcolm Granat, Andreas Holtermann, Kate Lyden

**Affiliations:** 1 School of Health and Society, University of Salford, Salford M6 6PU, UK; 2 National Research Centre for the Working Environment, Lersø Parkallé 105, 2100 Copenhagen, Denmark; 3 VivoSense, 27 Dorian, Newport Coast, CA 92657, USA

## 1. Introduction

The understanding and measurement of physical behaviours that occur in everyday life are essential not only for determining their relationship with health, but also for interventions, physical activity monitoring/surveillance of the population and specific groups, drug development, and developing public health guidelines and messages. Physical behaviour encompasses various activities and postures that are performed in response to different factors, domains and constraints, such as environmental and occupational factors, health-related factors, and personal choices. The fundamental components of free-living physical behaviour are the types of activities, their intensities, and their patterns in time. With the aid of body-worn sensors, we can now accurately identify and classify an increasing number of these activities (e.g., lying, sitting, standing, walking, cycling, and car transportation) and use advanced techniques to quantify their patterns. By understanding and quantifying these patterns, we can gain insights into behaviour at both individual and population levels, thereby transforming epidemiological and clinical research.

A Special Issue consisting of twelve high-quality papers has been published, comprising one systematic review, ten original research articles, and one experimental protocol for a pilot RCT. Of the ten research articles, three use machine learning techniques, five focus on new techniques for quantifying specific physical behaviours (e.g., lying, continuous walking bouts, sit-to-stand transitions, walking distance, and steps), one examines measuring orthotic wear, and the last explores an application for restraining night-time wandering in people with dementia.

## 2. Contributions

Mellema and Gjøvaag [1] conducted a scoping review to evaluate the purpose and reported outcome measures of wearable activity monitors in people with a lower limb amputation (LLA) in real-world situations. The review found that step count was the most frequently reported outcome measure, but caution is needed in choosing reliable outcome measures. The review also highlighted the limited capability of contemporary technology to provide a comprehensive picture of real-world ambulation in people with LLA. The authors call for further debate and definition of the framework of ecological validity in rehabilitation sciences and the development of wearable technologies to improve future studies of real-world ambulation in people with LLA.

The accuracy of machine learning activity classification models trained on laboratory-based activity trials under free-living conditions is low. To improve this accuracy, new models need to be trained on free-living accelerometer data and include shorter prediction windows, temporal features, such as standard deviation, and multiple sensors. Ahmadi et al. [2] used random forest (RF) activity classification models for preschool-aged children trained on free-living accelerometer data, which were evaluated. The results show that RF models provide accurate recognition of young children’s movement behaviours under real-world conditions, with the inclusion of temporal features improving accuracy, mainly for the wrist classification models.

Russell et al. [3] aimed to assess whether an AI model and single sensor measuring acceleration and ECG could model cognitive and physical fatigue for a self-paced trail run. The protocol involved inducing physical and cognitive fatigue in one healthy participant and collecting acceleration and ECG data using a single device. A fatigue prediction model was implemented using a convolutional neural network. Results showed that the AI model and single sensor could accurately measure cognitive and physical fatigue during a practical field protocol, including contextual factors. This has practical application to fatigue research in the field.

Limited data exist on how prosthetic devices are used to support lower-limb prosthesis users in their free-living environment, and there is a need to monitor a patient’s physical behaviour while using these devices. Griffiths et al. [4] developed a model for accurately classifying postures (sitting, standing, stepping, and lying) using data from a single shank-worn accelerometer. Data were collected from 14 anatomically intact participants and one amputee over three days. A random forest classifier with a 15 s window length provided the highest classification accuracy of 93% weighted average F-score and between 88 and 98% classification accuracy across all four posture classes, making it possible to embed an accelerometer-based activity monitor into the shank component of a prosthesis to capture physical behaviour information in both above and below-knee amputees. The models and software used in this study have been made open source to overcome the current restrictions of applying activity monitoring methods to lower-limb prosthesis users.

Body postural allocation during daily life is important for health, and thigh-worn accelerometers are useful for assessing it. However, existing algorithms to detect lying down from thigh-worn accelerometers are not usable across manufacturers. In a study by Hettiarachchi et al. [5], a refined algorithm based on thigh rotation, the standard deviation of acceleration, and time duration of sedentary bouts was developed and validated using Axivity-AX3 accelerometers. The refined algorithm demonstrated good agreement with the reference, indicating its potential for estimating lying time in studies using different accelerometer brands.

Defining a continuous walking event in free-living environments can be challenging due to interruptions, and no method has considered the intensity caused by these interruptions. Gbadamosi et al. [6] found that a novel method of combining short interruptions of time between walking events based on an average walking cadence increases the daily time spent in moderate-to-vigorous physical activity (MVPA). The average daily time spent in MVPA increased significantly using different walking cadence thresholds, indicating the potential for this method to enable future analyses of the associations between continuous walking and health-related outcomes.

Löppönen et al. [7] aimed to evaluate the day-to-day variability and year-to-year reproducibility of an accelerometer-based algorithm for sit-to-stand transitions in a free-living environment among community-dwelling older adults. Results showed that the volume and intensity of sit-to-stand transitions were reproducible from day to day and year to year, with high intraclass correlation coefficients. The study concludes that the accelerometer-based algorithm can be used to reliably study sit-to-stand transitions in free-living environments, which could be valuable in identifying individuals at increased risk for functional disability.

Measuring the total distance walked is crucial in tracking physical fitness and health status, but current methods can be subjective and time-consuming. Shah et al. [8] developed an algorithm, based on inertial sensors, to estimate the total distance walked by older adults and patients with multiple sclerosis. The algorithm calculates the distance travelled during each step and the total distance walked as the sum of walk distances for each stride, including turns. The proposed algorithm achieved good accuracy with respect to the manual, clinical standard, demonstrating the potential of wearable sensors in streamlining walk test administration. Further work is needed to test the generalizability of the algorithm with different administrators and populations.

Stepping-based physical activity targets are commonly used, and wrist-worn accelerometers are increasingly used for physical activity surveillance. However, the ability to derive stepping-based metrics from wrist-worn accelerometry lacks validation and open-source methods. Maylor et al. assessed the concurrent validity of two versions of the Verisense step-count algorithm at estimating [9] step-counts from wrist-worn accelerometry, in a population of 756 desk-based workers, compared with steps from the thigh-worn activPAL as the comparator. The optimized Verisense algorithm was found to be more accurate in detecting total and moderate-to-vigorous physical activity steps. The study highlights the importance of assessing algorithm performance beyond total step count and presents an acceptable accuracy for the derivation of stepping-based metrics from wrist-worn accelerometry.

Compliance with foot abduction brace (FAB) wear is important for the treatment of congenital clubfoot deformity, but self-reporting is time-consuming and inaccurate. Griffiths et al. [10] proposed a new method for objectively monitoring FAB wear using a single three-axis accelerometer. The method was tested on 11 families, and the measurements from the accelerometer and the physical diary were very similar, with a high level of agreement. The method has an advantage over existing objective monitoring solutions and can facilitate increased research into FAB compliance and help enable FAB monitoring in clinical practice.

Wandering is a common behavioural disorder among elderly people, and physical restraint is a common measure to address it, but it can lead to challenging behaviour in those with dementia. Cheung et al. [11] aimed to develop a virtual restraint using a night monitoring system (eNightLog) to provide a safe environment for the elderly and to reduce caregiver burden. The eNightLog system used remote sensors and an algorithm based on respiration rate and body posture to control an alarm system. The system’s accuracy and precision were compared to a pressure mat and an infrared fence system, and eNightLog demonstrated potential as an alternative to physical restraint.

Arguello et al. [12] present an experimental protocol for a 16-week pilot RCT, involving 46 participants, to investigate the influence of a virtual “companion” on enhancing exercise adherence among older adults. This companion supplements supervised exercise and employs a human-in-the-loop approach, wirelessly transmitted sensor-based activity measurement, and customized text messages to encourage behaviour change. They suggest that this may aid in developing a scalable hybrid AI companion to tackle the public health problem of sedentariness.

## 3. Conclusions

This special edition presents a comprehensive exploration of advancements in the understanding and measurement of free-living physical behaviours, demonstrating that the field extends far beyond merely measuring these behaviours to investigate their impact on human health. The collection of papers covers a wide range of topics, such as machine learning techniques, novel algorithms, and the use of wearable sensors in diverse populations, showcasing the growing potential of technology in this field.

Several studies emphasise the importance of refining algorithms and the innovative use of sensors for more accurate activity classification. Others focus on specific applications, including monitoring lower limb amputees, quantifying walking distance, and tracking compliance with orthotic wear. Furthermore, some papers explore the practical implementation of these methods, addressing the challenges of real-world ambulation and proposing solutions for individuals with specific needs, such as elderly individuals with dementia.

These developments have the potential to advance epidemiological and clinical research by providing more accurate, reliable, and user-friendly ways to assess physical behaviour and improve health outcomes. Consequently, they can contribute to more effective interventions, enhanced population surveillance, quantification of specific groups’ physical activity, and informed public health guidelines and messaging.

Further research is needed to build on these findings, refine the algorithms, and develop new wearable technologies for even more accurate and comprehensive monitoring of real-world ambulation and physical behaviours. By doing so, researchers and practitioners can collaborate to leverage technology in the pursuit of healthier, more active lifestyles for all.

## References

[1] Mellema M., Gjovaag T. (2022). Reported Outcome Measures in Studies of Real-World Ambulation in People with a Lower Limb Amputation: A Scoping Review. Sensors.

[2] Ahmadi M.N., Pavey T.G., Trost S.G. (2020). Machine Learning Models for Classifying Physical Activity in Free-Living Preschool Children. Sensors.

[3] Russell B., McDaid A., Toscano W., Hume P. (2021). Predicting Fatigue in Long Duration Mountain Events with a Single Sensor and Deep Learning Model. Sensors.

[4] Griffiths B., Diment L., Granat M.H. (2021). A Machine Learning Classification Model for Monitoring the Daily Physical Behaviour of Lower-Limb Amputees. Sensors.

[5] Hettiarachchi P., Aili K., Holtermann A., Stamatakis E., Svartengren M., Palm P. (2021). Validity of a Non-Proprietary Algorithm for Identifying Lying Down Using Raw Data from Thigh-Worn Triaxial Accelerometers. Sensors.

[6] Gbadamosi A.R., Griffiths B.N., Clarke-Cornwell A.M., Granat M.H. (2022). Defining Continuous Walking Events in Free-Living Environments: Mind the Gap. Sensors.

[7] Lopponen A., Karavirta L., Portegijs E., Koivunen K., Rantanen T., Finni T., Delecluse C., Roie E.V., Rantalainen T. (2021). Day-to-Day Variability and Year-to-Year Reproducibility of Accelerometer-Measured Free-Living Sit-to-Stand Transitions Volume and Intensity among Community-Dwelling Older Adults. Sensors.

[8] Shah V.V., Curtze C., Sowalsky K., Arpan I., Mancini M., Carlson-Kuhta P., El-Gohary M., Horak F.B., McNames J. (2022). Inertial Sensor Algorithm to Estimate Walk Distance. Sensors.

[9] Maylor B.D., Edwardson C.L., Dempsey P.C., Patterson M.R., Plekhanova T., Yates T., Rowlands A.V. (2022). Stepping towards More Intuitive Physical Activity Metrics with Wrist-Worn Accelerometry: Validity of an Open-Source Step-Count Algorithm. Sensors.

[10] Griffiths B., Silver N., Granat M.H., Lebel E. (2022). Measuring Foot Abduction Brace Wear Time Using a Single 3-Axis Accelerometer. Sensors.

[11] Cheung J.C., Tam E.W., Mak A.H., Chan T.T., Lai W.P., Zheng Y.P. (2021). Night-Time Monitoring System (eNightLog) for Elderly Wandering Behavior. Sensors.

[12] Arguello D., Rogers E., Denmark G.H., Lena J., Goodro T., Anderson-Song Q., Cloutier G., Hillman C.H., Kramer A.F., Castaneda-Sceppa C. (2023). Companion: A Pilot Randomized Clinical Trial to Test an Integrated Two-Way Communication and Near-Real-Time Sensing System for Detecting and Modifying Daily Inactivity among Adults > 60 Years-Design and Protocol. Sensors.

